# Exhausted olive pomace as a replacement for soybean hulls in corn silage–based diets for late-lactating Holstein cows: Insights from sustainable dairy farming

**DOI:** 10.3168/jdsc.2025-0932

**Published:** 2026-02-19

**Authors:** António J.M. Fonseca, Ana R.J. Cabrita

**Affiliations:** Network of Chemistry and Technology (REQUIMTE), Associated Laboratory for Green Chemistry (LAQV), School of Medicine and Biomedical Sciences (ICBAS), University of Porto, 4050-313 Porto, Portugal

## Abstract

This study evaluated the effects of replacing soybean hulls with exhausted olive pomace (EOP) on feed intake, milk production and composition, and rumen fermentation parameters. A cyclical changeover design experiment was used with 4 rumen-fistulated Holstein cows, 3 corn silage–based diets containing 0%, 3%, or 6% of EOP (DM basis), and three 21-d periods. Measurements were taken from d 15 to 21, and rumen contents were sampled on the last day. Diet did not affect feed intake, milk production and composition, or MUN. Ruminal pH, total short-chain fatty acid concentrations, and molar proportions of individual acids were also unaffected by the different dietary treatments. These findings suggest that EOP comprises a sustainable feed resource for late-lactating dairy cows fed corn silage–based diets, supporting circular economy approaches to promote the compound feed industry's resilience, competitiveness, and sustainability.

## Highlights

• EOP is a byproduct of the olive oil industry.
• Effects of EOP in dairy cow feeding are unknown.
• EOP can successfully replace soybean hulls in corn silage-based diets.
• No effects were observed on rumen fermentation parameters and animal performance.
• EOP contributes to a more sustainable food-chain production.

## Graphical Abstract

**Figure:**
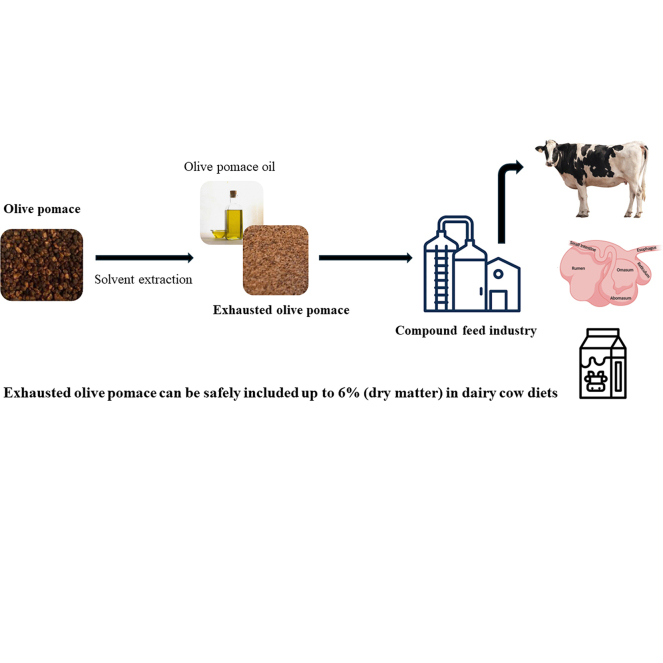
**Summary:** The olive oil industry, largely dominated by the southern countries of the European Union, generates substantial quantities of olive pomace as a byproduct. Dried olive pomace retaining appreciable ether extract can be processed with food-grade solvents to recover residual oil, yielding olive pomace oil and exhausted olive pomace (EOP). This study evaluated the effects of replacing soybean hulls with EOP in corn silage-based diets on feed intake, milk yield and composition, and rumen fermentation in late-lactating dairy cows. Dietary inclusion of EOP up to 6% of dry matter did not affect feed intake, milk production, milk composition, ruminal pH, total short-chain fatty acid concentration, or individual fatty acid molar proportions. These findings demonstrate that EOP is a viable, sustainable feed ingredient for late-lactation dairy cows fed corn silage-based diets, supporting circular economy strategies and enhancing the resilience, competitiveness, and sustainability of the compound feed industry worldwide.

The European Union compound feed industry is predominantly dependent (75%) on imported high-protein feed sources from nonmember countries (FEFAC, 2024). In Portugal, this industry faces an even more pronounced structural constraint, given its dependence on imported raw materials for approximately 80% of its inputs. This interdependence renders the sector particularly vulnerable to the volatility of international markets, logistics costs, and geopolitical disruptions, thereby affecting the competitiveness of the broader national livestock sector (IACA, 2025). In this scenario, it is imperative to identify opportunities within the framework of the circular economy that facilitate a gradual transition to a more autonomous, circular, and sustainable production model. This model should also be based on the valorization of agricultural and agro-industrial byproducts that the country produces in large quantities and that have yet to be extensively explored as raw materials for the compound feed industry, particularly for ruminant animals.

The olive oil industry is globally dominated by the European Union and is an important part of the agricultural economy in southern countries. Spain, Italy, and Greece are the largest producers, but current projections by the Association of Olive Growers and Wine Presses of Portugal, based on a consortium study conducted by independent consultants, indicate that Portugal is expected to become the third largest European producer of olive oil within the next few years (Olivum, 2024). Over the last 4 marketing years, Portuguese productivity has increased considerably more than that of other primary producing countries. Most olive mills currently operate under a 2-phase extraction system, in which a decanter separates the olive oil from wet olive pomace that due to its high moisture content (65%–75%) is dried after being partly destoned. The dried pomace that still has a reasonable ether extract (**EE**) content may undergo extraction with food-grade solvent, usually hexane, to recover residual oil, yielding olive pomace oil. The resulting defatted partly destoned olive pomace is known as exhausted olive pomace (**EOP**), which is mainly used for bioenergy production through direct combustion, gasification, and pyrolysis. However, these disposal strategies introduce environmental concerns, such as the release of particulate matter and GHG emissions, and increased energy consumption (Enaime et al., 2024).

The expected increase in EOP availability, its content of organic and bioactive compounds, and its low cost, combined with the need for more sustainable livestock production systems, support the interest in moving to a waste to resource concept under a circular economy approach. This is particularly important for ruminants, which are naturally efficient at utilizing “low-grade, non-human edible by-products” generated by the circular economy (FEFAC, 2022, p. 1). Over the years, several studies have evaluated olive byproducts as dietary ingredients for ruminants, including lactating ruminants, with most focusing on full-fat olive pomace (Faye et al., 2013; Terramoccia et al., 2013; Scicutella et al., 2023), which is relatively high in energy and dietary unsaturated lipids from olive oil. However, to our knowledge, no previous studies have evaluated the inclusion of EOP as a dietary ingredient in dairy cows. Indeed, EOP typically has low nutritional value due to its high NDF and ADL content, low EE content, and limited fiber digestibility and energy value, and its use has traditionally been restricted to supplementation for ruminants raised under rainfed extensive systems during periods of feed shortage (FEDNA, 2021).

We hypothesized that EOP could replace soybean hulls (**SH**), an imported feed resource, as a sustainable feed source in corn silage–based diets for late-lactating Holstein cows without compromising productivity, providing insights from sustainable dairy farming and supporting a circular economy approach to dairy nutrition. This study assessed its effects on performance and rumen fermentation parameters. A direct replacement of half and the total SH with EOP in diet formulation was chosen to avoid confounding effects that may arise when multiple raw materials are changed simultaneously in the compound feed, resulting in inclusion levels of 3% and 6% (DM basis), more likely to be applied under practical feeding conditions.

The experiment was conducted between November 2024 and January 2025 at the Vairão Agricultural Campus of the School of Medicine and Biomedical Sciences, University of Porto (**ICBAS-UP**; Vila do Conde, Portugal), and all procedures involving animals were approved by the Animal Ethics Committee of the ICBAS-UP, and licensed by the Portuguese General Directorate of Food and Veterinary Affairs (permit #0421/000/000/2021). Four third-lactation Holstein cows fitted with a 10-cm-diameter rumen cannula (Bar Diamond Inc., Parma, ID) were used, averaging 286 DIM (SD = 19.8) and 27 kg/d of milk (SD = 4.2). The cows were housed in adjacent individual boxes ranging from 10 to 11.5 m^2^, which encouraged contact, grooming, and socializing. The cows had continuous access to drinking water and mineral salt blocks. The cows had access to an outdoor paddock from 1000 to 1230 h. The animals were assigned to dietary treatment sequences in a cyclical changeover design experiment. Treatments were randomly assigned across cows and periods so that each animal received all treatments once. Each experimental period lasted for 21 d, with measurements performed from d 15 to 21. Experimental diets contained (DM basis) 58% corn silage, 7% chopped barley straw, and 35% concentrate. A common concentrate mixture was formulated with 17% of SH included in the control diet. Increasing dietary levels of EOP (3% and 6%, DM basis) were achieved by replacing half or all of SH with EOP (Aucama–Agro Industrial Valpacense, Lda, Valpaços, Portugal). The experimental diets were named according to their EOP content: 0% (**EOP0**), 3% (**EOP3**), and 6% (**EOP6**; Table 1). Diets were fed as TMR for ad libitum intake, with fresh feed offered each day on 2 equal meals (0900 and 1830 h). Orts were collected and weighed daily after the morning milking. The amount of feed offered was adjusted weekly to maintain approximately 5% refusals. Samples of experimental feeds and orts were collected on 3 nonconsecutive days during each measurement period for chemical composition analysis. Cows were milked twice daily at 0800 and 1800 h. Milk production was measured throughout the experimental period. Milk was sampled at both milkings on 2 consecutive days during the final week of each measurement period and analyzed for fat, protein, lactose, and MUN by mid-infrared spectroscopy (Fossomatic + MilkoScan 7 RM, Foss Analytical, Hiller⊘d, Denmark). Milk composition data were corrected according to the proportion of milk yield from each milking relative to the total daily yield. On the last day of each experimental period, rumen contents were collected 4 h after the morning feeding. The pH was measured immediately, and subsamples were frozen at −20°C for later analysis.

**Table 1:** Ingredients and chemical composition of the experimental diets (as-offered), and exhausted olive pomace (EOP)

Item	Diet^1^			EOP
	EOP0	EOP3	EOP6	
Ingredient (% of DM)				
Corn silage	58	58	58	
Chopped barley straw	7.0	7.0	7.0	
Wheat grain	5.6	5.6	5.6	
Rapeseed meal	7.0	7.0	7.0	
Soybean meal	7.7	7.7	7.7	
Sunflower meal	5.6	5.6	5.6	
Soybean hulls^2^	6.0	3.0	—	
Exhausted olive pomace	—	3.0	6.0	
Cane molasses	1.0	1.0	1.0	
Calcium carbonate	0.74	0.74	0.74	
Calcium soaps	0.35	0.35	0.35	
Urea	0.17	0.17	0.17	
Sodium bicarbonate	0.31	0.31	0.31	
Magnesium oxide	0.21	0.21	0.21	
Mineral and vitamin premix^3^	0.14	0.14	0.14	
Salt	0.18	0.18	0.18	
Chemical composition (% of DM, except for DM content)				
DM (%)	47.2	47.2	47.2	89.5
Ash	6.00	6.15	6.16	5.48
CP	13.6	14.2	14.4	11.9
EE	2.80	2.87	3.03	3.28
NDF	36.4	36.9	36.3	73.1
ADF	22.6	22.7	22.6	50.8
ADL	5.44	5.74	6.46	45.6
Starch	24.8	24.1	24.1	—
P	0.35	0.36	0.40	0.19

1 Diets are named according to their EOP content: EOP0 = 0%, EOP3 = 3%, and EOP6 = 6%.

2 According to the analytical quality control of the compound feed manufacturer, the chemical composition determined by near-infrared spectroscopy was as follows (DM basis): 5.12% ash, 12.1% CP, 2.11% EE, and 41.3% crude fiber.

3 Contained: 5,000,000 IU/kg vitamin A, 750,000 IU/kg vitamin D_3_, 20,000 mg/kg vitamin E, 375 mg/kg vitamin B_6_, 1,313 mg/kg vitamin C, 500 mg/kg β-carotene, 13,310 mg/kg betaine anhydrous, 2,500 mg/kg Fe, 17,500 mg/kg Zn, 5,000 mg/kg Cu, 15,000 mg/kg Mn, 450 mg/kg I, 98 mg/kg Co, 15 mg/kg Se, and 75 mg/kg organic Se.

Ground samples (1 mm) of experimental raw materials, corn silage, barley straw, and concentrate mixtures were analyzed in duplicate according to AOAC International (2000) for DM (method 934.01), ash (method 942.05), EE (method 920.39), and Kjeldahl N (method 954.01). Crude protein was calculated as Kjeldahl N × 6.25 (method 954.01). Neutral detergent fiber, ADF, and ADL were determined by the detergent procedures of Robertson and Van Soest (1981) and Van Soest et al. (1991). During NDF extraction, α-amylase was added except for barley straw; sodium sulfite was not added. Neutral detergent fiber was expressed without residual ash. Starch was analyzed in ground samples (0.5 mm) using the method described by Salomonsson et al. (1984). Phosphorus was determined by spectrophotometry, according to Commission Regulation (EC) No. 152/2009 (Annex III, Part P; European Commission, 2009), based on ISO 6491 (ISO, 1998).

To determine the total and individual short-chain fatty acid (**SCFA**) concentrations in rumen fluid, 1.00-mL aliquots were mixed with 250 µL of 25% wt/wt H_3_PO_4_, homogenized, and centrifuged (13,680 × *g*, 20 min, 4°C). Next, 250 µL of supernatant was transferred to 1.5-mL GC vials containing 250 µL of ultrapure water and 500 µL of internal standard solution (1.00 mmol/L, 3-methylvaleric acid). Samples were homogenized before GC-flame ionization detection (**FID**) analysis. Calibration was performed using 5 standards similarly, replacing the sample with 250 µL of a mixture 25% wt/wt H_3_PO_4_, water (1:4, vol/vol) and 250 µL of SCFA standard mixtures; the internal standard was identical. Correlation coefficients (r) > 0.999 were required. A GC-FID system (Shimadzu GC-2010 Plus, Shimadzu Corporation, Kyoto, Japan) featuring a capillary column HP-FFAP (19091F-443), 30 m × 0.25 mm, 0.25 µm (Agilent Technologies, Santa Clara, CA), was used. Injection volume was 1 µL (split 1:50); injector and FID temperatures were 260°C. The oven program was 100°C (5 min), then 6°C/min to 220°C (5 min). Helium was used as the carrier gas. Total run was 30 min. The retention times were acetic acid, 8.8 min; propionic acid, 10.8 min; *iso*-butyric acid, 11.4 min; butyric acid, 12.8 min; *iso*-valeric acid, 13.7 min; valeric acid, 15.2 min; and caproic acid, 17.4 min.

Data were analyzed using the MIXED procedure of SAS OnDemand for Academics (2025, SAS Institute Inc., Cary, NC). The mixed model included the fixed effects of period and diet, the random effect of cow, and the residual error. Because the diet effect was never significant, no further analyses were carried out.

Experimental diets differed only on the replacement of SH by EOP up to 6% (DM basis), presenting a similar chemical composition. Crude protein, NDF, and starch contents averaged approximately 14.1%, 36.5%, and 24.4% (DM basis), respectively (Table 1). Although the EOP was partly destoned, it presented a high content of lignified cell wall components (73% and 46% for NDF and ADF fractions, respectively), a moderate CP content (12%), and a residual EE content of 3.3%. As refusals for late-lactation cows are often recommended to be lower than for early-lactation animals, a target refusal level of 5% was selected to ensure that cows were fed ad libitum and to obtain results applicable under on-farm conditions. Treatment effects on DMI, milk production, milk composition, and feed efficiency are presented in Table 2. No diet effect was observed for DMI and milk production that averaged 24.6 and 25.2 kg/d, respectively. Similarly, the replacement of SH with EOP kept unaffected milk composition, including MUN. Feed efficiency measured as milk production or ECM per DMI was also not affected by diet. Ruminal fermentation parameters, including pH, total SCFA concentration, and molar proportions of individual acids, were also not affected by the different dietary treatments (Table 3).

**Table 2:** Least squares means for feed intake, milk production and composition, and feed efficiency from the different dietary treatments

Item	Diet^1^			SEM	*P*-value
	EOP0	EOP3	EOP6		
DMI (kg/d)	24.6	24.3	24.8	0.84	0.880
Yield (kg/d)					
Milk	24.9	25.0	25.6	1.38	0.918
ECM^2^	28.2	29.1	29.1	1.67	0.723
Fat	1.15	1.21	1.21	0.098	0.258
Protein	1.02	1.03	1.02	0.046	0.977
Lactose	1.19	1.20	1.21	0.075	0.975
Composition (%)					
Fat	4.64	4.83	4.76	0.395	0.690
Protein	4.09	4.10	4.00	0.154	0.455
Lactose	4.77	4.78	4.73	0.094	0.884
MUN (mg/dL)	11.3	12.0	11.4	0.549	0.331
Milk/DMI	1.01	1.04	1.03	0.060	0.720
ECM/DMI	1.14	1.20	1.18	0.054	0.110

1 Diets are named according to their exhausted olive pomace (EOP) content: EOP0 = 0%, EOP3 = 3%, and EOP6 = 6%.

2 ECM = 12.51 × kg of fat + 7.41 × kg of CP + 5.32 × kg of lactose (NASEM, 2021).

**Table 3:** Least squares means for ruminal fermentation parameters from the different dietary treatments

Item	Diet^1^			SEM	*P*-value
	EOP0	EOP3	EOP6		
pH	5.81	6.10	6.02	0.129	0.359
Total SCFA^2^ (mmol/L)	145.8	130.3	140.8	5.84	0.150
SCFA profile (% mol)					
Acetate	60.8	63.8	62.0	1.87	0.269
Propionate	23.7	19.9	21.9	2.03	0.234
*Iso*-butyrate	0.73	0.83	0.81	0.062	0.528
Butyrate	11.0	11.5	11.9	0.75	0.468
*Iso*-valerate	1.75	2.04	1.88	0.159	0.413
Valerate	1.52	1.38	1.24	0.150	0.171
Caproate	0.40	0.56	0.30	1.38	0.209
Acetate/propionate	2.67	3.34	2.86	0.349	0.221
Propionate/butyrate	2.30	1.72	1.86	0.313	0.254

1 Diets are named according to their exhausted olive pomace (EOP) content: EOP0 = 0%, EOP3 = 3%, and EOP6 = 6%.

2 SCFA = short-chain fatty acids.

The present study evaluated the effects of replacing SH with EOP in a common complementary compound feed formula for corn silage–based TMR on dairy cow performance, along with the impact of dietary EOP on rumen fermentation parameters. The chemical composition of EOP is influenced by olive variety, cultivation practices, fruit ripeness, and the technologies used for virgin olive oil extraction and for pomace olive oil production, among other factors (Molina-Alcaide and Yáñez-Ruiz, 2008). Cell wall polysaccharides and lignin were the main constituents of the EOP studied, with low residual EE and a moderate P content. Despite some variation, particularly the higher ADL content, our results are consistent with previous studies (Kotsampasi et al., 2017; Paié-Ribeiro et al., 2024). As lignin is the main constituent that limits the feed value of olive pomace, a pretreatment with alkaline hydrogen peroxide was proposed as a nontoxic technology to enhance rumen digestibility of EOP (Masmoudi et al., 2024).

Evaluation of olive byproducts as animal feed has been focused on olive pomace with high fat content that it is being reported to keep unaffected milk yield while shifting milk fatty acid composition toward higher proportion of unsaturated fatty acids. Indeed, Scicutella et al. (2023) showed that destoned and fresh olive pomace from a 2-phase milling process (20.6% EE, DM basis) can be successfully fed to dairy cows, as it does not affect either protein and NDF degradability or animal performance, modulating the biohydrogenation toward the improvement of beneficial fatty acid content, increasing the nutritional quality of milk. Castellani et al. (2017) investigated the effects of dietary supplementation with dried olive pomace (15.2% EE, DM basis) on milk yield and composition of dairy cows and concluded that this strategy enhanced the nutritional and nutraceutical properties of milk, while also modifying the aroma of both milk and the cheese produced. Similarly, inclusion of olive pulp (7.9% EE, DM basis) in the concentrate of Murciano-Granadina dairy goats did not affect rumen function or milk yield, but increased milk protein content and improved the fatty acid profile, particularly CLA (Sánchez-García et al., 2025).

Conversely, studies evaluating EOP effects on animal performance are scarce. In the present study, the direct replacement of SH with EOP did not affect any measured parameter. Despite the known differences in energy value between SH and EOP, which were likely reflected in the dietary ratio of potentially fermentable ME to RDP, MUN concentrations were not affected. Indeed, as the EOP3 and EOP6 diets should have lower fermentable ME:RDP ratios than EOP0, higher MUN concentrations would be expected (Cabrita et al., 2003). Olive pomace is characterized by high concentrations of total phenolic compounds, *ortho*-diphenols, flavonoids, total tannins, and condensed tannins, making it a promising source of value-added bioactive compounds (Contreras et al., 2021; Paié-Ribeiro et al., 2024). Notably, Paié-Ribeiro et al. (2024) reported higher concentrations of total phenols, *ortho*-diphenols, and flavonoids, which were associated with higher antioxidant activity in EOP samples than olive pomace samples obtained by pressing or centrifugation. Contreras et al. (2021) demonstrated that, among other phenolic compounds, EOP was a source of hydroxytyrosol and its derivatives, compounds known for their antioxidant activity. Moreover, phenolic compounds have been considered as a strategy to decrease methanogenesis as specific rumen bacteria can reduce them with hydrogen or formate consumption and transforming into acetate, an energy-yielding product for the host (Krumholz and Bryant, 1986). However, effects are known to be dependent on the nature of the phenolic compound (Romero et al., 2023). In the present study, no effects were observed on SFCA profile. Similarly, Scicutella et al. (2023) did not find effects on ruminal acetate content, but significantly decreased propionic, butyric, and *iso*-valeric acid content when lactating dairy cows were fed a diet with 8% (DM basis) of olive oil pomace with 20.8% of EE (DM basis).

Similar to the absence of effects on animal performance observed in the present study, Kotsampasi et al. (2017) concluded that using EOP (5.5% EE, DM basis) as a dietary supplement for male growing lambs did not affect their performance or carcass characteristics, but improved qualitative traits of the carcass. Additionally, Nunes et al. (2024) demonstrated that including EOP (2% EE, DM basis) in the diet of growing New Zealand × Californian rabbits (35 to 66 d old) can successfully replace traditional fiber sources without compromising animal performance or visceral development.

Despite the well-recognized importance of using byproducts from the agricultural industry in animal feeds, studies on effects of EOP on rumen function and animal performance are almost nonexistent. Findings of the present study suggest that EOP supplementation up to 6% (DM basis) does not compromise feed intake or milk production and composition of late-lactating dairy cows fed a corn silage–based TMR. For a more sustainable food chain production and alignment with increasing availability and low cost, integrative studies combining animal performance with microbial, biochemical, and omics-based analyses are needed for a complete understanding of EOP effects.
